# Health-related quality of life in men and women who experienced cardiovascular diseases: Tehran Lipid and Glucose Study

**DOI:** 10.1186/s12955-021-01861-2

**Published:** 2021-09-26

**Authors:** Sara Jalali-Farahani, Parisa Amiri, Hanieh Fakhredin, Kiana Torshizi, Leila Cheraghi, Davood Khalili, Fereidoun Azizi

**Affiliations:** 1 Research Center for Social Determinants of Health, Research Institute for Endocrine Sciences, Shahid Beheshti University of Medical Sciences, P.O. Box: 19395-4763, Tehran, Islamic Republic of Iran; 2 Students’ Research Committee, Shahid Beheshti University of Medical Sciences, Tehran, Islamic Republic of Iran; 3 Dalla Lana School of Public Health, University of Toronto, Toronto, Canada; 4 Department of Epidemiology and Biostatistics, Research Institute for Endocrine Sciences, Shahid Beheshti University of Medical Sciences, Tehran, Islamic Republic of Iran; 5 Prevention of Metabolic Disorders Research Center, Research Institute for Endocrine Sciences, Shahid Beheshti University of Medical Sciences, Tehran, Islamic Republic of Iran; 6 Endocrine Research Center, Research Institute for Endocrine Sciences, Shahid Beheshti University of Medical Sciences, Tehran, Islamic Republic of Iran

**Keywords:** Cardiovascular diseases, Health-related quality of life, Adults, Tehran lipid and glucose study

## Abstract

**Background:**

Cardiovascular diseases (CVDs) are among the most common causes of death worldwide, including in Iran. Considering the adverse effects of CVDs on physical and psychosocial health; this study aims to investigate the association between experience of CVDs and health-related quality of life (HRQoL) in adult participants of the Tehran Lipid and Glucose Study (TLGS).

**Methods:**

The participants of this cross-sectional study were 7009 adults (≥ 20 years) who participated in the TLGS during 2014–2017. Demographic information and HRQoL data was collected through validated questionnaires by trained interviewers. HRQoL was assessed by the Iranian version of the SF-12 questionnaire. Data was analyzed using the SPSS software.

**Results:**

The mean age of participants was 46.8 ± 14.6 years and 46.1% of them were men. A total of 9.0% of men and 4.4% of women had CVDs. In men, the mean physical HRQoL summary score was significantly lower in those with CVDs compared to those without CVDs (46.6 ± 0.8 vs. 48.5 ± 0.7, *p* > 0.001). In women, the mean mental HRQoL summary scores was significantly lower in those with CVDs compared to those without CVDs (42.8 ± 1.0 vs. 45.2 ± 0.5, *p* = 0.009). In adjusted models, men with CVDs were more likely to report poor physical HRQoL compared to men without CVDs (OR(95%CI): 1.93(1.32–2.84), *p* = 0.001); whereas for women, the chance of reporting poor mental HRQoL was 68% higher in those with CVDs than those without CVDs (OR(95%CI): 1.68(1.11–2.54), *p* = 0.015).

**Conclusion:**

The findings of the current study indicate poorer HRQoL in those who experienced CVDs compared to their healthy counterparts with a sex specific pattern. While for men, CVDs were associated with more significant impairment in the physical dimension of HRQoL, women experienced a similar impairment in the mental dimension of HRQoL.

## Introduction

Cardiovascular diseases (CVDs) are among the most common causes of deteriorating health and death worldwide. In 2015, a total of 17.9 million deaths and 347.5 million disability-adjusted life years (DALYs) in the world were owing to CVDs [1, 2]. It is estimated that by 2030, about 30.5% (more than 23 million) of all global deaths will be due to CVDs [3]. While there is a declining trend in the mortality rates of CVDs in high-income countries, about half of global CVD mortality occurred in low and middle-income countries, especially in the Eastern Mediterranean countries [2]. Similarly, in Iran, the most common causes of death have shifted from infectious diseases to non-communicable diseases (NCDs) in recent decades [4]. Based on existing evidence, Iran was found to have some of the highest prevalence of CVDs [5]. In addition to the high prevalence of CVDs and their economic burden on families and the healthcare system [6], their related outcomes such as stroke, myocardial infarction (MI) and coronary artery disease (CAD) can lead to fatal and non-fatal complications. Although therapeutic advances have contributed to a reduction in the mortality rate of cardiovascular outcomes, many patients of CVDs still experience other complications throughout their lives, such as stress, anxiety, fatigue and pain, sleep disturbances, shortness of breath and dyspnea [7]. In more serious cases, complications can include heart failure, the possibility of recurrent MI and sudden death [8–13]. These complications have important consequences on individuals’ mental and physical health, namely, their health-related quality of life (HRQoL).

HRQoL is a multidimensional concept that involves subjective evaluations of physical, mental, and social domains of one’s health [14]. Improving HRQoL is the ultimate goal of all health-related interventions [15]. In recent decades, the increasing proportion of individuals suffering from NCDs has been accompanied by greater interest in evaluating and improving the quality of life in individuals with these conditions [16]. In this regard, many studies found negative associations between cardiovascular outcomes and physical and mental dimensions of HRQoL [9, 17, 18]. Several factors influenced the amount of impairment such as individual’s age, employment, depression and anxiety, the duration of the disease, severity of angina, inadequate social support, complications of therapies and the experience of serious consequences such as heart failure [19–22].

Although the association between CVDs and HRQoL has been well documented in previous studies around the world [9, 17–20, 22–25], most of these studies did not explicitly consider sex differences [9, 17–20, 22] with only three studies conducting sex-specific analyses [23–25]. The association between CVDs and HRQoL has also been investigated in previous studies conducted in Tehran [26–28] and other cities of Iran [29–33]. However, most of the previous studies have only focused on a specific population of people with CVDs such as those who experienced either heart failure, myocardial infarction, ischemic heart disease or had undergone a coronary artery bypass graft [9, 13, 22–28, 34]. There is a lack of sex-specific evidence comparing HRQoL in a general population of adults with and without CVDs incidence. Furthermore, the studies previously conducted in Tehran had small sample sizes, and only one study addressed sex differences [26]. Due to limited evidence exploring this association in an Iranian population, the current study has two aims: first, to investigate the association between CVDs and the HRQoL in a large sample of Tehranian adults who participated in a cohort of the Tehran Lipid and Glucose Study (TLGS); and second, to explore any sex-specific differences in the association between CVDs and HRQoL. We hypothesized that participants with CVDs will demonstrate a poorer HRQoL compared to their healthy counterparts, which would follow a sex specific pattern.

## Methods

### Study design and participants

This cross sectional study was conducted within the framework of the Tehran Lipid and Glucose Study (TLGS), which is a community-based study that begun in 1999 and continued for 20 years. The main objectives of the TLGS were to identify risk factors of non-communicable diseases and factors associated with preventing them. A total of 15,005 individuals aged ≥ 3 years who were residence of district No.13 of Tehran, were selected and participated in the TLGS. Further details regarding rational and design of the TLGS have been published previously [35, 36]. This study has been approved by the ethics committee of the Research Institute for Endocrine Sciences (RIES) of Shahid Beheshti University of Medical Sciences and all participants provided written informed consent. For the current analysis, data from all adults (≥ 20 years) who had participated in the 6th phase of the TLGS (2014–2017) and had complete information on HRQoL and CVDs incident were considered. Of 7939 individuals aged ≥ 20 years with complete HRQoL data who participated in the 6th phase of the TLGS, 930 participants were excluded due to having missing information on confounding variables; finally, data from 7009 eligible participants was analyzed. According to the results of a similar previous study [23], the minimum required sample size to compare the physical and mental HRQoL scores between the two groups of participants with and without CVDs estimated to be 907 people. Considering estimated sample size, the 7009 eligible particpants of the current study is sufficient.

### Measurements and instruments

Participants' weights were measured and recorded with minimal clothing, without shoes, using a digital scale with an accuracy of 0.1 kg. Height measurement was performed by a tape in a standing position, without shoes and while the shoulders were in a normal position. Body mass index (BMI) was calculated using related formula as participant’s weight in kilograms divided by the square of height in meters. Using BMI, participants were stratified into three weight status: (1) Normal weight: BMI < 25 kg/m^2^, (2) Overweight: BMI of 25 to < 30 kg/m^2^, and (3) Obese: BMI ≥ 30 kg/m^2^). Data on demographic information, physical activity, and HRQoL were collected using valid questionnaires and through interviews. Demographic information included age, sex (male/female), marital status (Single/Divorced/Widowed/Married), level of education (Primary was defined as having education less than high school diploma/Secondary was defined as earning high school diploma/Higher was defined as earning any academic degree), and employment status (Unemployed/Student/Housewife/Unemployed, but had other sources of income/Employed) of participants. Physical activity was evaluated by the Persian version of the Modifiable Activity Questionnaire (MAQ). Based on the available evidence, the Persian version of MAQ has acceptable validity and reliability [37]. In this questionnaire, physical activity was assessed through the number of times and the duration of time that the individual partakes in physical activity in a typical week. Level of physical activity was calculated using metabolic equivalents (MET) minutes/week, and the level of participants’ physical activity was grouped into either low (MET < 600 min/week), moderate (600 ≤ MET < 3000 min/week) or high activity (MET ≥ 3000 min/week) [38].

HRQoL was assessed by the SF-12 questionnaire. The questionnaire assesses health status using eight subscales including physical functioning, role physical, bodily pain, general health, vitality, social function, role emotional, and mental health. The range of scores for each scale is from zero to 100, with zero indicating the worst and 100 indicating the best position on each subscale. The psychometric characteristics of this questionnaire have been studied in the Iranian adult population with favorable validity and reliability [39].

Hypertension (HTN) was defined as SBP and/or DBP more than 140 and 90 mm/Hg, respectively, based on joint national committee criteria [40]; Type 2 diabetes was defined as fasting blood sugar FBS ≥ 126 mg/dl or 2-h post-load glucose ≥ 200 mg/dl or taking medication for diagnosed diabetes [41]; chronic kidney disease (CKD) was defined as structural or functional kidney damage or GFR < 60 ml/min/1.73 m^2^ present for more than three months according to the Kidney Disease Outcome Quality Initiative (K/DOQI) guidelines [42]. History of cancer includes those with any report of cancer during their life.

In the current study, CVD was defined as any measures of CHD events in recent 18 years plus stroke or cerebrovascular events. CHD events are defined as cases of (1) definite myocardial infarction diagnosed by ECG and biomarkers, (2) probable myocardial infarction (positive ECG findings plus cardiac symptoms or signs but biomarkers showing negative or equivocal results), (3) unstable angina pectoris (new cardiac symptoms or changing symptom patterns and positive ECG findings with normal biomarkers), (4) angiographic‐proven CHD, and (5) CHD death; these are comparable with ICD 10 ruric I20-I25. Stroke was defined as a new neurological deficit that lasts more than 24 h (ICD 10 rubric I60-I69). Details of the definitions and analysis of CVD outcome data have been described before [43].

### Statistical analysis

The distribution of continues variables was checked graphically. For all variables, the distribution diagram was drawn and compared with the normal distribution diagram. Normal continuous variables were expressed as mean ± sd, while medians (Q1–Q3) were reported for skewed variables. Frequencies and percentages were reported for categorical variables. Distribution of variables among groups was compared using independent samples T-test, Mann–Whitney and Chi-square test as appropriate. The HRQoL scores were compared between study participants with and without CVDs using analysis of covariance, for men and women separately. For assessing the association between HRQoL and CVDs status (absent vs. present), unadjusted and adjusted logistic regression analyses were used. Poor HRQoL was defined as the first tertile of physical component summary (PCS) or mental component summary (MCS). The odds ratios (ORs) and 95% confidence intervals (CIs) were reported, separately for men and women. All variables which were significantly different between the study participants with and without CVDs were adjusted. A two-sided *P* value < 0.05 was deemed statistically significant. Statistical analyses were conducted using IBM SPSS 22 software for Windows (IBM Co., Armonk, NY, USA).

## Results

The mean age of participants was 46.8 ± 14.6 years and 46.1% of them were men. A total of 457 (6.5%) of participants had CVDs. Descriptive statistics of study participants are shown in Table 1. There were significant differences in the distribution of marital status, level of education, job status, levels of physical activity and smoking status between men and women. Lower percentage of women (27.6%) had low levels of physical activity compared to men (37.5%); however, higher percentages of men (23.9%) had high levels of physical activity compared to women (12.2%). In terms of smoking, a significantly higher percentage of men were smokers compared to women (42.1% vs. 5.4%, respectively). In terms of cardiovascular risk factors, women had significantly higher mean body mass index (BMI), total cholesterol, and high-density lipoproteins (HDL) compared to men (*p* < 0.001). On the other hand, men had significantly higher fasting blood sugar, triglycerides, and systolic and diastolic blood pressure than women. The distribution of weight status in men and women was also significantly different, with a higher prevalence of overweight in men and higher prevalence of obesity in women. Hypertension and CVDs were significantly higher in men than in women, while chronic kidney diseases were significantly higher in women than men.

**Table 1:** Descriptive statistics of the study participants

	Total (n = 7009)	Men (n = 3232)	Women (n = 3777)	Values*	*P* value
Age (year)	46.8 ± 14.6	46.9 ± 15.3	46.7 ± 13.9	0.59^a^	0.558
Marital status					
Single/divorced/widowed	1570 (22.4)	665 (20.6)	905 (24.0)	11.5^b^	< 0.001
Married	5439 (77.6)	2567 (79.4)	2872 (76.0)		
Level of education					
Primary	1692 (24.1)	635 (19.6)	1057 (28.0)	70.2^b^	< 0.001
Secondary	2837 (40.5)	1349 (41.7)	1488 (39.4)		
Higher	2480 (35.4)	1248 (38.6)	1232 (32.6)		
Job status					
Unemployed/student/housewife	2887 (41.2)	218 (6.7)	2669 (70.7)	3005.4^b^	< 0.001
Unemployed, but had other sources of income	978 (14.0)	603 (18.7)	375 (9.9)		
Employed	3144 (44.9)	2411 (74.6)	733 (19.4)		
Physical activity					
Low	2256 (32.2)	1213 (37.5)	1043 (27.6)	352.2^b^	< 0.001
Moderate	3520 (50.2)	1246 (38.6)	2274 (60.2)		
High	1233 (17.6)	773 (23.9)	460 (12.2)		
Smoking					
Yes	1563 (22.3)	1360 (42.1)	203 (5.4)	1354.2^b^	< 0.001
No	5446 (77.7)	1872 (57.9)	3574 (94.6)		
Cardio-metabolic risk factors					
Body mass index (kg/m^2^)	28.1 ± 4.9	27.5 ± 4.4	28.4 ± 5.3	− 9.10^a^	< 0.001
Fasting blood sugar	98.8 ± 28.3	100.5 ± 28.9	97.3 ± 27.6	4.78^a^	< 0.001
Total cholesterol	186.1 ± 39.3	183.5 ± 38.7	188.4 ± 39.6	− 5.21^a^	< 0.001
HDL	47.1 ± 11.2	42.6 ± 9.6	50.8 ± 11.0	− 33.2^a^	< 0.001
Triglyceride	123 (86.0–175.0)	135 (95.0–192.0)	114 (80.0–160.0)	− 13.6^c^	< 0.001
Systolic blood pressure	114.7 ± 16.5	118.4 ± 15.3	111.5 ± 16.9	18.1^a^	< 0.001
Diastolic blood pressure	76.1 ± 9.8	78.5 ± 9.6	74.0 ± 9.5	19.1^a^	< 0.001
Body weight status					
Normal weight	1918 (27.4)	913 (28.2)	1005 (26.6)	100.9^b^	< 0.001
Overweight	2979 (42.5)	1531 (47.4)	1448 (38.3)		
Obese	2112 (30.1)	788 (24.4)	1324 (35.1)		
Diabetes (yes)	1048 (15.0)	474 (14.7)	574 (15.2)	0.39^b^	0.534
Hypertension (yes)	1577 (22.5)	769 (23.8)	808 (21.4)	5.76^b^	0.016
CVD (yes)	457 (6.5)	292 (9.0)	165 (4.4)	62.2^b^	< 0.001
Chronic kidney diseases (yes)	1630 (23.3)	502 (15.5)	1128 (29.9)	200.5^b^	< 0.001
Cancer (yes)	88 (1.3)	34 (1.1)	54 (1.4)	2.0^b^	0.157

Data are reported as mean ± SD for normal continuous variables and medians (Q1–Q3) for skewed variables. n (%) were reported for categorical variables. *Values are t-value (a), χ^2^ (b) and z value (c)

Table 2 shows the mean scores of HRQoL in men and women. In all subscales, men had significantly higher scores compared to women. Similarly, men had significantly higher physical and mental summary scores compared to women.

**Table 2:** Comparison of health-related quality of life scores in men and women

	Total (n = 7009)	Men (n = 3232)	Women (n = 3777)	t value	*P* value
Physical function	83.9 ± 24.9	88.9 ± 20.7	79.7 ± 27.2	16.1	< 0.001
Role physical	79.7 ± 23.0	86.6 ± 19.3	73.9 ± 24.2	24.5	< 0.001
Bodily pain	78.9 ± 24.0	84.4 ± 20.7	73.8 ± 25.4	20.0	< 0.001
General health	48.1 ± 23.1	50.9 ± 23.2	45.6 ± 22.7	9.6	< 0.001
PCS	48.4 ± 8.4	50.2 ± 7.3	46.9 ± 8.9	16.9	< 0.001
Vitality	64.5 ± 25.8	69.8 ± 24.2	59.9 ± 26.2	16.5	< 0.001
Social function	81.3 ± 25.9	85.1 ± 23.5	78.0 ± 27.3	11.8	< 0.001
Role emotional	75.2 ± 23.3	80.5 ± 21.2	70.7 ± 24.0	18.1	< 0.001
Mental health	69.9 ± 22.1	74.8 ± 20.9	65.7 ± 22.3	17.5	< 0.001
MCS	48.4 ± 10.9	50.6 ± 10.3	46.6 ± 11.1	15.6	< 0.001

*PCS* physical component summary, *MCS* mental component summary

Data are reported as mean ± SD. *P* values were derived using t-test

Descriptive statistics of participants based on incident of CVDs are presented in Table 3. As it is indicated, except for smoking and BMI in men and history of cancer in women, all other variables were significantly different in those with and without CVDs outcomes. Therefore, these variables were adjusted in all analyses in Table 4 and in all regression models.

**Table 3:** Descriptive statistics in men and women based on CVDs

	Men (n = 3232)		Value*	*P* value	Women (n = 3777)		Value*	*P* value
	Without CVDs (n = 2940)	With CVDs (n = 292)			Without CVDs (n = 3612)	With CVDs (n = 165)		
Age (year)	45.1 ± 14.6	65.0 ± 10.2	− 30.3^a^	< 0.001	45.9 ± 13.6	64.9 ± 7.9	− 28.9^a^	< 0.001
Marital status								
Single/divorced/widowed	653 (22.2)	12 (4.1)	53.3^b^	< 0.001	851 (23.6)	54 (32.7)	7.28^b^	0.007
Married	2287 (77.8)	280 (95.9)			2761 (76.4)	111 (67.3)		
Level of education								
Primary	516 (17.6)	119 (40.8)	104.4^b^	< 0.001	932 (25.8)	125 (75.8)	200.6^b^	< 0.001
Secondary	1232 (41.9)	117 (40.1)			1454 (40.3)	34 (20.6)		
Higher	1192 (40.5)	56 (19.2)			1226 (33.9)	6 (3.6)		
Job status								
Unemployed/student/housewife	213 (7.2)	5 (1.7)	310.3^b^	< 0.001	2556 (70.8)	113 (68.5)	108.0^b^	< 0.001
Unemployed, but had other sources of income	437 (14.9)	166 (56.8)			324 (9.0)	51 (30.9)		
Employed	2290 (77.9)	121 (41.4)			732 (20.3)	1 (0.6)		
Physical activity								
Low	1098 (37.3)	115 (39.4)	6.03^b^	0.049	975 (27.0)	68 (41.2)	19.3^b^	< 0.001
Moderate	1122 (38.2)	124 (42.5)			2186 (60.5)	88 (53.3)		
High	720 (24.5)	53 (18.2)			451 (12.5)	9 (5.5)		
Smoking								
Yes	1233 (41.9)	127 (43.5)	0.26^b^	0.608	200 (5.5)	3 (1.8)	4.3^b^	0.038
No	1707 (58.1)	165 (56.5)			3412 (94.5)	162 (98.2)		
Body mass index (kg/m^2^)	27.5 ± 4.4	27.3 ± 3.8	0.87^a^	0.384	28.4 ± 5.3	30.9 ± 4.9	− 5.89^a^	< 0.001
Diabetes (yes)	353 (12.0)	121 (41.4)	183.8^b^	< 0.001	499 (13.8)	75 (45.5)	122.6^b^	< 0.001
Hypertension (yes)	601 (20.4)	168 (57.5)	201.5^b^	< 0.001	694 (19.2)	114 (69.1)	233.4^b^	< 0.001
Chronic kidney diseases (yes)	381 (13.0)	121 (41.4)	164.2^b^	< 0.001	1004 (27.8)	124 (75.2)	168.9^b^	< 0.001
Cancer (yes)	26 (0.9)	8 (2.7)	8.78^b^	0.009	51 (1.4)	3 (1.8)	0.19^b^	0.510

Data are reported as mean ± SD for continues variables and n(%) for categorical variables. *P* values for normal continuous variables were achieved using t-test. For categorical variables, chi-square test was used. *Values are t-value (a) and χ^2^(b)

**Table 4:** Mean health-related quality of life scores based on cardiovascular diseases (CVDs) outcomes in men and women

	Men (n = 3232)		*P* value	Women (n = 3777)		*P* value
	Without CVDs	With CVDs		Without CVDs	With CVDs	
Physical function	84.1 ± 1.9	78.3 ± 2.2	< 0.001	78.7 ± 1.2	75.0 ± 2.3	0.084
Role physical	83.3 ± 1.8	78.1 ± 2.1	< 0.001	73.4 ± 1.1	68.0 ± 2.1	0.003
Bodily pain	85.2 ± 2.0	83.2 ± 2.2	0.146	74.8 ± 1.1	72.4 ± 2.2	0.263
General health	45.4 ± 2.0	41.0 ± 2.3	0.003	43.2 ± 1.0	39.5 ± 1.9	0.033
PCS	48.5 ± 0.7	46.6 ± 0.8	< 0.001	47.2 ± 0.4	45.9 ± 0.7	0.058
Vitality	69.8 ± 2.3	64.3 ± 2.6	0.001	60.6 ± 1.2	52.9 ± 2.3	< 0.001
Social function	84.2 ± 2.2	83.6 ± 2.5	0.706	75.6 ± 1.3	69.3 ± 2.4	0.006
Role emotional	79.2 ± 2.0	76.4 ± 2.2	0.014	68.5 ± 1.1	64.4 ± 2.1	0.040
Mental health	75.4 ± 2.0	74.6 ± 2.2	0.575	62.3 ± 1.0	58.5 ± 2.0	0.045
MCS	51.0 ± 0.9	50.4 ± 1.1	0.349	45.2 ± 0.5	42.8 ± 1.0	0.009

Adjusted mean and standard error have been reported (Adjustments in men: age, level of education, job status, marital status, physical activity and diabetes, hypertension, cancer and chronic kidney diseases, and in women: age, level of education, job status, marital status, physical activity, smoking, body mass index and diabetes, hypertension and chronic kidney diseases)

*PCS* physical component summary, *MCS* mental component summary

Comparison of HRQoL scores between groups of participants with and without CVDs are indicated in Table 4. All subscale scores of HRQoL were significantly lower in those with CVDs compared to those without CVDs, except for bodily pain, social function and mental health subscales in men and physical function and bodily pain subscales in women. The PCS in men and MCS in women were significantly lower in those with CVDs compared to those without CVDs.

Table 5 reports adjusted odds ratios and 95% confidence intervals (CIs) of poor physical and mental HRQoL for men and women with and without CVDs. In men, the chances of reporting poor physical HRQoL were significantly higher in those with CVDs compared to those without CVDs in both unadjusted (OR (95%CI): 4.27 (3.02–6.04), *p* < 0.001) and adjusted models (OR (95%CI):1.93 (1.32–2.84), *p* = 0.001). However, there was no significant difference in the chances of reporting poor mental HRQoL in men based on CVDs incidence, in both unadjusted (OR (95%CI): 0.76 (0.57-1.02), *p*=0.068) and adjusted models (OR (95%CI): 1.21 (0.87-1.68), *p*=0.270). On the other hand, in unadjusted models in women, the chances of reporting poor HRQoL were significantly higher in those with CVDs compared to those without CVDs in both physical (OR (95%CI): 3.54 (2.31–5.44), *p* < 0.001) and mental (OR (95%CI): 1.49(1.01–2.20), *p* = 0.043) aspects of HRQoL; however, after adjusting for confounding variables, only the chance of reporting poor mental HRQoL was significantly higher in women with CVDs compared to those without CVDs (OR (95%CI): 1.68 (1.11–2.54), *p* = 0.015).

**Table 5:** Adjusted odds ratios and 95% confidence interval (CI) for poor physical and mental health-related quality of life in men and women

	PCS		MCS	
	Odds ratio	*P* value	Odds ratio	*P* value
*Men (n = 3232)*				
CVDs (yes)	1.93 (1.32–2.84)	**0.001**	1.21 (0.87–1.68)	0.270
Age	1.02 (1.01–1.03)	**0.003**	0.98 (0.97–0.99)	**0.002**
Marital status				
Married	Ref		Ref	
Unmarried	0.54 (0.41–0.71)	**< 0.001**	1.46 (1.12–1.90)	**0.005**
Level of education				
Higher	Ref		Ref	
Secondary	1.05 (0.86–1.29)	0.632	1.24 (1.02–1.52)	**0.030**
Primary	1.72 (1.29–2.29)	**< 0.001**	1.05 (0.80–1.38)	0.714
Job status				
Employed	Ref		Ref	
Unemployed, but had other sources of income	1.02 (0.75–1.39)	0.889	1.06 (0.80–1.42)	0.671
Unemployed/student/housewife	0.90 (0.55–1.49)	0.690	1.07 (0.67–1.70)	0.788
Physical activity levels				
High	Ref		Ref	
Moderate	0.81 (0.64–1.03)	0.088	1.51 (1.20–1.90)	**< 0.001**
Low	1.27 (0.99–1.61)	0.054	1.42 (1.13–1.79)	**0.003**
Diabetes (yes)	1.78 (1.32–2.39)	**< 0.001**	1.24 (0.95–1.61)	0.120
Hypertension (yes)	1.31 (1.04–1.67)	**0.022**	0.88 (0.70–1.10)	0.249
Chronic kidney diseases (yes)	1.02 (0.77–1.36)	0.875	0.76 (0.58–0.99)	**0.040**
Cancer (yes)	2.69 (1.01–7.17)	**0.048**	0.78 (0.33–1.86)	0.581
*Women (n = 3777)*				
CVDs (yes)	1.06 (0.66–1.70)	0.799	1.68 (1.11–2.54)	**0.015**
Age	1.05 (1.04–1.06)	**< 0.001**	0.98 (0.97–0.99)	**0.001**
Marital status				
Married	Ref		Ref	
Unmarried	0.53 (0.41–0.69)	**< 0.001**	1.27 (1.01–1.59)	**0.039**
Level of education				
Higher	Ref		Ref	
Secondary	1.18 (0.93–1.50)	0.185	1.60 (1.29–1.99)	**< 0.001**
Primary	1.38 (1.01–1.90)	**0.046**	1.97 (1.48–2.63)	**< 0.001**
Job status				
Employed	Ref		Ref	
Unemployed, but had other sources of income	0.68 (0.46–1.02)	0.065	1.49 (1.03–2.16)	**0.033**
Unemployed/student/housewife	0.82 (0.57–1.18)	0.277	1.36 (0.98–1.89)	0.069
Physical activity levels				
High	Ref		Ref	
Moderate	1.37 (1.04–1.82)	**0.028**	1.09 (0.85–1.40)	0.480
Low	2.18 (1.59–2.98)	**< 0.001**	1.17 (0.89–1.53)	0.269
Smoking (yes)	1.08 (0.72–1.60)	0.715	2.01 (1.38–2.92)	**< 0.001**
BMI	1.08 (1.06–1.10)	**< 0.001**	1.01 (0.99–1.02)	0.592
Diabetes (yes)	1.26 (0.96–1.65)	0.090	0.90 (0.71–1.14)	0.381
Hypertension (yes)	1.04 (0.80–1.34)	0.794	1.21 (0.96–1.52)	0.106
Chronic kidney diseases (yes)	0.75 (0.60–0.94)	**0.011**	0.93 (0.75–1.14)	0.455

Data in bold, indicate statistically significant* p*-values

*PCS* physical component summary, *MCS* mental component summary

## Discussion

The present study aimed to investigate the association between CVDs and HRQoL in Tehranian men and women who participated in the TLGS. The findings indicate that HRQoL scores were significantly lower in participants with CVDs incident compared to those without CVDs. In addition, a sex-specific pattern was observed for the association between CVDs and HRQoL. While the impairment of HRQoL scores was observed in mental dimensions of HRQoL in women; in men, this impairment was more pronounced in physical dimension of HRQoL.

According to the findings of the current study, HRQoL scores were significantly lower in those with history of CVDs compared to those without history of CVDs. Consistent with our findings, several studies in different countries reported impairments in HRQoL in patients who experienced CVD outcomes compared to their healthy counterparts [9, 12, 23, 25, 44, 45]. Similarly, findings of a study conducted in Tehran, Iran, indicated HRQoL scores in all physical and mental subscales were significantly lower in men and women who suffered from MI compared to healthy individuals, with physical subscales more impaired than mental ones [46]. Experiencing CVDs is often accompanied with several health consequences such as limitations in physical function, physical disabilities, decreased social interactions, psychological distress such as anxiety and stress, decreased vitality, early retirement due to inability to work, pain and fatigue, shortness of breath, and sleep disturbances; all of which can negatively impact various aspects of HRQoL [13, 47–49].

In the current study, a sex specific pattern was observed in the association between CVDs and HRQoL. In terms of HRQoL subscale scores, impairment of HRQoL in men with CVDs was more prominent in physical subscales; while in women with CVDs, lower HRQoL scores were observed in all mental subscales and to less extent in physical subscales compared to their counterparts without CVDs. There were greater impairments in HRQoL in women compared to men. One possibility for this sex difference may be due to lower compatibility with disease and slower recovery from illnesses in women in comparison to men, ultimately leading to more impairments in HRQoL [50, 51]. Another explanation for lower HRQoL scores could be related to factors such as age, psychosocial characteristics, and baseline health-related quality of life scores which have been found to be important predictors of HRQoL in CVD survivors [22]. Existing evidence indicate that women develop CVDs in older age due to the cardioprotective effect of estrogen in their reproductive stage of life; moreover, they suffer from depression more often than men, and had lower HRQoL scores compared to their male counterparts [52, 53]. Furthermore, another study found that social support is a significant determinant of HRQoL in female cardiac patients specifically in the mental dimension of HRQoL [54]. In the TLGS general population, perceived social support from family was significantly lower in women compared to men [55]. If social support is a significant determinant of HRQoL, it makes sense that women in this study experienced lower HRQoL than men, who perceived greater social support from family in their lives. A male dominant society as well as more exposure to support services in terms of social and physical activities may provide another explanation for less impairment of mental HRQoL in men compared to women [56].

Furthermore, in the current study, the chances of reporting poor physical HRQoL in men and poor mental HRQoL in women were significantly higher in those with CVDs compared to their counterparts. Related existing evidence has indicated that mood disorders, psychosomatic and psychological symptoms have been reported more in women with cardiovascular outcomes [57] compared to men, which may exacerbate the mental dimension of HRQoL in women. Despite the higher prevalence of myocardial infarction in men, women appear to have a similar or slightly higher prevalence of stable angina [58]. Studies have shown that women are more likely to have non-obstructive coronary artery disease, whereas men have more obstructive coronary artery disease and multivessel involvement in angiographic studies than women in the population referred with acute coronary syndrome [59, 60]. These findings justify the reduction of invasive therapeutic interventions in women and the lower risk of developing refractory angina and rehospitalization for unstable angina and ultimately improving their prognosis [53, 61]. On the other hand, following the higher prevalence of MI in men, they are more likely to have HFrEF (heart failure with reduced ejection fraction). But regardless of its type either HFpEF (Heart failaure with preserved ejection fraction) or HFrEF (Heart failaure with reduced ejection fraction), women showed to have a better therapeutic response, maybe because compensatory responses at the cellular or molecular level appear to be more effective in women than in men [59, 62] which may contribute to the lower score of physical HRQoL in men. In addition, chronic obstructive pulmonary disease (COPD) is strongly associated with CVDs, which is more prevalent in men compared to women [63, 64]. Considering the impairment of the PCS in the presence of comorbidities [65]; the COPD may be another factor that contributed to the experiencing less physical function and more physical limitations in male participants of the current study with CVDs compared to their female counterparts.

Of the strengths of this study is using precise detections of CVD outcomes which allows for more accurate findings and interpretations. In addition, the use of the SF-12 questionnaire, one of the most common and popular tools for assessing HRQoL in general populations, make the findings of this study more directly comparable to those of other countries that use the same questionnaire. This study also has limitations related to its generalizability and design. First, the cross-sectional design of the study precludes causal inferences in the relationship between CVDs and HRQoL. Second, the participants of this study were all residents of Tehran, a large urban city; hence, the findings cannot be generalized to broader rural or sub-urban communities in Iran. In addition, in the current study, because the estimated effect sizes of the group-based comparisons were small, the significant differences observed in HRQoL scores between those with and without CVD outcomes may be due to the large sample size and should be interpreted with caution. Moreover, data of income was not collected in the current study. Since, income may influence the HRQoL of particpants; therefore, in future studies, it is recommended to collect participants’ income and include it in future analysis. Lastly, the SF-12 assesses general health status and may not be as responsive as a disease-specific instrument for CVDs. However, as a generic measurement, it allows for comparisons of HRQoL impairments across a range of clinical conditions, and healthy individuals. Considering coincidence of CVDs with other diseases, i.e., diabetes, hypertension, chronic kidney diseases; we have just adjusted effects of these conditions in our analysis. However, it would be valuable to make comparisons between CVDs and mentioned conditions in future studies.

## Conclusions

Findings of the current study indicate a significant association between CVDs and HRQoL with a sex specific pattern. In men, CVDs are associated with an impairment in the physical dimension of HRQoL, while in women, of the association was evident for mental dimensions of HRQoL. These findings can help to better plan and design interventions and the distribution of health care resources to improve HRQoL in people with CVDs incidence.

## Data Availability

The datasets used and/or analyzed during this study are available from the corresponding author on reasonable request.
